# Plasma Cytokine Levels in Fibromyalgia and Their Response to 15 Weeks of Progressive Resistance Exercise or Relaxation Therapy

**DOI:** 10.1155/2018/3985154

**Published:** 2018-04-18

**Authors:** M. Ernberg, N. Christidis, B. Ghafouri, I. Bileviciute-Ljungar, M. Löfgren, J. Bjersing, A. Palstam, A. Larsson, K. Mannerkorpi, B. Gerdle, E. Kosek

**Affiliations:** ^1^Department of Dental Medicine, Karolinska Institutet and Scandinavian Center for Orofacial Neurosciences (SCON), 141 04 Huddinge, Sweden; ^2^Pain and Rehabilitation Centre and Department of Medical and Health Sciences, Linköping University, 581 85 Linköping, Sweden; ^3^Department of Clinical Sciences, Karolinska Institutet and Department of Rehabilitation Medicine, Danderyd Hospital, 182 88 Stockholm, Sweden; ^4^Department of Rheumatology and Inflammation Research, Institute of Medicine, Sahlgrenska Academy, University of Gothenburg, 405 30 Göteborg, Sweden; ^5^Institute of Neuroscience and Physiology, Section of Health and Rehabilitation, Physiotherapy, Sahlgrenska Academy, University of Gothenburg, 405 30 Göteborg, Sweden; ^6^University of Gothenburg Centre for Person Centred Care (GPCC), Sahlgrenska Academy, 405 30 Göteborg, Sweden; ^7^Department of Clinical Neuroscience, Karolinska Institutet, 171 77 Stockholm, Sweden; ^8^Stockholm Spine Center, 194 89 Stockholm, Sweden

## Abstract

The aims of this study were to compare circulating cytokines between FM and healthy controls and to investigate the effect on cytokine levels by 15 weeks of progressive resistance exercise or relaxation therapy in FM. Baseline plasma cytokine levels and clinical data were analyzed in 125 women with FM and 130 age-matched healthy women. The FM women were then randomized to progressive resistance exercise (*n* = 49) or relaxation (*n* = 43). Baseline IL-2, IL-6, TNF-*α*, IP-10, and eotaxin were higher in FM than in healthy controls (*P* < 0.041), whereas IL-1*β* was lower (*P* < 0.001). There were weak correlations between cytokine levels and clinical variables. After both interventions, IL-1ra had increased (*P* = 0.004), while IL-1*β* had increased in the relaxation group (*P* = 0.002). Changes of IFN-*γ*, IL-2, IL-4, IL-6, IL-8, and IL-17A were weakly correlated with changes of PPT, but there were no significant correlations between changes of cytokine and changes in other clinical variables. The elevated plasma levels of several cytokines supports the hypothesis that chronic systemic inflammation may underlie the pathophysiology of FM even if the relation to clinical variables was weak. However, 15 weeks of resistance exercise, as performed in this study, did not show any anti-inflammatory effect on neither FM symptoms nor clinical and functional variables. This trial is registered with ClinicalTrials.gov NCT01226784, registered October 21, 2010. The first patient was recruited October 28, 2010.

## 1. Introduction

Fibromyalgia (FM) is a pain disorder that is characterized by widespread pain and tenderness, stiffness, fatigue, sleep disturbances, and cognitive dysfunction [1]. The prevalence is approximately 2-3%, which means that as many as 25 million people may be affected in Europe. FM peaks around 55–65 years of age and shows a strong female predominance with 80–90% of cases being women [2]. The consequences for the individual and the society are physical and psychological distress, loss of work productivity, reduced quality of life, and increased use of health resources. There are no objective tests to diagnose FM which is why the diagnosis traditionally has been based on history and clinical examination of tender points [3]. In 2010, new criteria, purely based on history, were proposed [4]. These criteria were originally regarded as provisional, but have since then been modified and are now recommended for clinical use by the American College of Rheumatology [5].

Despite its huge impact for the individual and the society, the mechanisms behind altered nociception in FM are not well known, but it is currently believed that interactions between the autonomic nervous system, the hypothalamus-pituitary-adrenal axis, and the immune system are of importance [6]. The autonomic nervous system controls blood flow and as FM patients seem to have an increased sympathetic nervous system activity and hyporeactivity to various stressors, including physical activity [7], muscle ischemia with ensuing release of inflammatory substance may develop. Increased sympathetic tone has been related to pain and associated with the increased release of the cytokine interleukin-8 (IL-8) [8]. Thus, an inflammatory/stress feedback dysregulation and an augmented release of inflammatory cytokines from peripheral blood cells, such as monocytes and neutrophils, may be an underlying cause of FM [9]. In support of this hypothesis is that symptoms, generally attributed to release of cytokines in inflammatory conditions, such as fatigue, hyperalgesia, and allodynia, are key symptoms also in FM [10]. However, previous studies have not demonstrated a consistent blood cytokine pattern in FM. A systematic review with meta-analysis concluded a few years ago that patients with FM have elevated blood levels of IL-1 receptor antagonist (IL-1ra), IL-6, and IL-8. On the other hand, study heterogeneity was great and the quality of included studies in general was low [11]. Since then, few studies have reported elevated plasma levels of, for example, IL-8, monocyte chemoattractant protein-1 (MCP-1) [12], and IL-17A [13], but reduced levels of IL-4, IL-5, and IL-13 [14]. A recent study reported increased levels of several proteins, including IL-8 in cerebrospinal fluid (CSF) and plasma in FM compared to blood donors and healthy controls [15]. Studies have further reported increased release of IL-1*β*, tumor necrosis factor alpha (TNF-*α*), IL-6, and IL-10 from stimulated monocytes in FM [16]. However, other studies have reported that mitogen stimulation of cultured blood mononuclear cells from patients with FM led to reduced release of IL-6, IL-8, and MCP-1 compared to healthy individuals and patients with autoimmune diseases [17, 18]. To summarize, there is still debate whether peripheral cytokine levels are altered in FM.

Regular exercise of moderate intensity is recommended in most management programs for FM [19]. Moderate- to high-intensity physical exercise not only has beneficial health effects in general, but it also activates endogenous pain inhibitory pathways, which is why it may relieve FM symptoms. Indeed, the beneficial effects of regular exercise in FM have been demonstrated in several studies [20–22], including our recent randomized controlled multicenter study [23], which showed improvement in muscle function, health status, and pain intensity. Garcia-Hermoso and coworkers in a systematic review [21] reported that moderate-intensity exercise (aerobic and aquatic exercise) performed for 30–60 minutes at least two times per week increased functional aerobic capacity and favored the activities of daily living with FM. Another systematic review by Bidonde et al. [22] found positive results of diverse exercise interventions on pain, multidimensional function, and self-reported physical function. However, intolerance to exercise due to deficient pain inhibition is one of the key symptoms in FM [24] and many patients experience worsening of symptoms for a few days after exercise, which may be attributed to increased release of metabolic and inflammatory substances in peripheral tissue [25]. On the contrary, after a longer period of exercise, alterations in circulating cytokine levels have been reported in FM and a systematic review recently concluded that, despite minimal evidence, exercise interventions might act as an anti-inflammatory treatment by reducing IL-6 and IL-8 levels [26]. Further, in FM patients subjected to 15 weeks of Nordic walking, changes of insulin growth factor-1 (IGF-1) in serum correlated positively to changes of pain threshold, indicating a possible beneficial role for IGF-1 during exercise [27].

This study had two aims: first was to compare plasma levels of cytokines between FM and healthy controls and their relation to clinical variables and second was to investigate the effect in FM on cytokine levels by a progressive resistance exercise or relaxation intervention in relation to clinical variables.

## 2. Methods

### 2.1. Study Design and Participants

**Figure 1:**
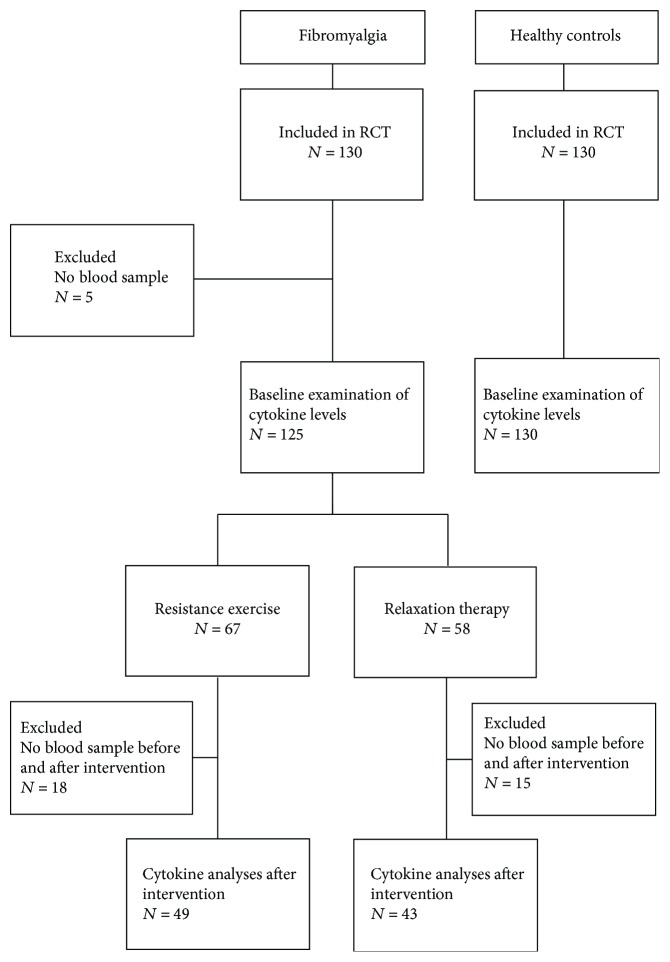
Flow chart of the participants included in the study.

The study is a substudy of a randomized controlled multicenter trial regarding the effects of progressive resistance exercise in 130 women with FM (http://Clinicaltrials.gov NCTO1226784). The enrollment process for the FM women has been described in detail previously [23]. For baseline comparisons, 130 age-matched healthy women were included. A flow chart of the participants in this study is shown in Figure 1.

Inclusion criteria for both groups were to be of working age (20–65 years). For the FM women, a diagnosis of FM according to the ACR-1990 classification criteria [3] was also required, and for the controls good general health and no current pain.

Exclusion criteria for both groups were (1) high blood pressure (>160/90 mmHg), (2) osteoarthritis in the hip or knee, (3) other severe somatic or psychiatric disorders, (4) primary causes of pain other than FM, (5) high consumption of alcohol (audit > 6), (6) participation in a rehabilitation program within the past year, (7) regular resistance exercise or relaxation therapy twice a week or more, (8) inability to understand or speak Swedish, and (9) not being able to refrain from analgesics, NSAID, or hypnotics for 48 hours prior to examinations.

The study was conducted at three centers in Sweden (Gothenburg, Linkoping, and Stockholm) and in accordance with the Helsinki Declaration and Good Clinical Practice. The Central Ethical Review Board in Stockholm approved the study (Dnr: 2010/1121-31/3). All participants received verbal and written information about the study and gave their written consent. They were compensated economically for their participation.

### 2.2. Procedure

Participants (both FM and healthy women) who responded to newspaper advertisements were telephone screened for possible eligibility. If they were willing to participate they were scheduled for medical examination by an experienced physician to verify that they were eligible. For all participants age (years) and blood pressures (mm Hg) were registered, in addition to weight (kg) and height (m) to calculate body mass index (BMI). Current medication and alcohol consumption (Audit) was registered. A venous blood sample (20 mL) was drawn from the decubital vein. For the FM women, a diagnosis of FM according to the ACR 1990 criteria was verified [3], and pain duration as well as year of FM diagnosis was recorded. Participants who were included were scheduled for baseline assessments about one week thereafter. During this visit, pain intensity, pain disability, health aspects, and psychological distress was assessed by questionnaire, physical capacity (functional tests) by a physiotherapist, and number of tender points and pressure algometry by a physician or physiotherapist as described previously [23].

FM women were then randomized to either progressive resistance exercise or relaxation therapy (active control). Randomization was performed in blocks of 6 subjects by a computer-generated sequence (http://www.randomization.com) separately for each of the participating sites by a researcher not involved in data sampling (ME). For each participant, the treatment was concealed in sequentially numbered, sealed, opaque envelopes.

One week after the 15-week intervention was finished, pain variables, physical activity, psychological distress, and quality of life were again assessed, and physical capacity, number of tender points, and pressure algometry were recorded. Finally, a venous blood sample was drawn.

### 2.3. Psychometric Instruments

A 0–100 mm visual analogue scale (VAS) with the endpoints 0 = no pain and 100 = worst imaginable pain was used for assessment of global pain intensity. Pain disability was assessed with the pain disability index (PDI). The instrument measures the impact that pain has on the ability of a person to participate in essential life activities on a scale from 0 to 70 [28]. The participants also answered the Short Form Health Survey (SF36) that assesses health-related quality of life; a higher score indicates better health. From the SF36, the physical (SF36-PSC) and mental (SF36-MSC) summary components were calculated and used as background variables. Further, the Swedish versions of the fibromyalgia impact questionnaire (FIQ) and the multidimensional fatigue inventory (MFI) were completed. FIQ is comprised of ten subscales of disabilities and symptoms ranging from 0 to 100. The total score is the mean of ten subscales and a higher score indicates a lower health status [29]. The MFI is used to assess five different dimensions of fatigue: general fatigue, physical fatigue, reduced motivation, reduced activity, and mental fatigue and it consists of 20 questions [30]. For assessment of psychological distress, the validated Swedish versions of the hospital anxiety and depression scale (HADS) and the Pain Catastrophizing Scale (PCS) [31].

### 2.4. Physical Capacity

Physical capacity was measured using four tests: hand grip force, maximal isometric elbow flexion force, isometric knee extension force, and a 6 min walk test (6MWT) [23].

Maximal hand grip force (N) was recorded bilaterally with the Grippit® device (AB Detektor, Göteborg, Sweden). The mean force over a set period of time (ten seconds) was recorded and the best performance out of two trials (with one minute rest between each trial) was used.

Maximal isometric elbow flexion force (kg) was measured bilaterally with a dynamometer (Isobex®; Medical Device Solutions AG, Oberburg, Switzerland). The participant was seated without back support with legs stretched out in the front. The upper arm was aligned with the trunk and the elbow was bent in 90° flexion. The maximum force obtained during a period of 5 seconds was recorded and the best performance out of three trails was used.

Static knee extension force (N) was recorded bilaterally with a dynamometer (Steve Strong®; Stig Starke HBI, Göteborg, Sweden). The participant was seated in a fixed position with back support and the knee and hip in 90° of flexion and legs hanging freely. A nonelastic strap was placed around the ankle and attached to a pressure transducer with an amplifier. The maximum force obtained during a period of 5 seconds was recorded and the best performance out of three trails was used.

The 6MWT was determined by measuring the distance that the participant could walk during six minutes in a standardized situation.

For hand grip force, maximal isometric elbow flexion force, and isometric knee extension force, the average force for the right side was calculated and used in the analyses.

### 2.5. Pressure Algometry

To get an estimate of the participants general pain sensitivity [32], pressure pain thresholds (PPTs) were recorded with an electronic algometer (Somedic Sales AB, Höör, Sweden). The algometer has a blunt rubber tip of 1 cm^2^, and a pressure rate of 30 kPa/s PPT was used. The PPTs were recorded bilaterally over 4 points. To avoid the risk of temporal summation, each site was assessed only once. The same order was used for all participants, starting with points on the right side in the following order: the supraspinatus muscle (at origins above the scapula spine near the medial border), the lateral epicondyle (2 cm distal to the epicondyles), the gluteus maximus (in upper outer quadrants of buttocks in anterior fold of muscle), and the inside of the knee (at the medial fat pad proximal to the joint line). The algometer was held perpendicular to the skin at the site, and subjects were instructed to press the button when the PPT was reached, that is, as soon as the sensation of pressure changed to pain. When a maximum pressure of 1500 kPa was reached, the application of pressure ceased [23]. The mean value of all eight recordings was used; that is, the PPT was recorded with the individual as the basis.

### 2.6. Resistance Exercise Intervention

During the progressive resistance exercise, the participants exercised twice per week during 15 weeks under the supervision of specially trained physical therapists, either at physiotherapy premises or at a local gym. The program aimed to improve muscle strength and health status and included exercises improving core stability and power. It was adopted from a previous study that used a similar protocol to investigate the effect of resistance exercise on pain and functional capacity in FM [33]. As loading muscles of upper extremities was expected to increase the risk of activity-induced pain, more emphasis was given on the lower extremities. Each session started with 10 min bicycling to warm up and was then followed by a 50 min strength training protocol, focusing on the lower extremities. The training was initiated at low loads at 40% of the maximum voluntary capacity (MVC) and successively progressed up to 70–80% of MVC [23].

### 2.7. Relaxation Therapy

Relaxation therapy was chosen as active control as it is often integrated in multidisciplinary rehabilitation for patients with FM [34]. The relaxation therapy was performed at physiotherapy premises twice per week during 15 weeks and was guided by experienced physiotherapists. Autogenic training, which refers to a series of mental exercises including relaxation and autosuggestion, was used. The physiotherapist guided the participants through their bodies by focusing their minds on the bodily experience of relaxation and letting the body part in focus rest on the ground. The duration of the relaxations therapy was approximately 25 minutes and was thereafter followed by stretching exercises [23].

### 2.8. Handling of Samples and Biochemical Analyses

The blood samples were placed on ice and immediately transported to the laboratory where they were centrifuged (1500*g*) for 30 min. The plasma was pipetted into 1.5 mL Eppendorf vials and frozen (−70 ° C) until analyses.

Plasma samples were analyzed for several pro- and anti-inflammatory cytokines and chemokines (IFN-*γ*, IL-1*β*, IL-2, IL-4, IL-6, IL-8, IL-10, IL-17A, TNF-*α*, IL-1ra, eotaxin, IFN-γ-inducible protein 10 (IP-10), and MCP-1). Luminex technology (Bio-Plex, Bio-Rad Laboratories Inc., Hercules, CA, USA) using standard kits with magnetic beads was used. To be able to detect cytokines with low plasma concentration, a high-sensitivity kit (HSCYTMAG-60SK, Merck KGaA, Darmstadt, Germany) was used for most cytokines. For IL-1ra, eotaxin, IP-10, and MCP-1, a high-sensitivity kit was not available which is why another standard kit was used (HCYTOMAG-60K, Merck KGaA, Darmstadt, Germany). The limits of detection were for IFN-*γ* < 0.48 pg/mL, for IL-1*β* < 0.14 pg/mL, for IL-2 < 0.19 pg/mL, for IL-4 < 1.12 pg/mL, for IL-6 < 0.11 pg/mL, for IL-8 < 0.13 pg/mL, for IL-10 < 0.56 pg/mL, for IL-17A < 0.33 pg/mL, for TNF-*α* < 0.16 pg/mL, for IL-1ra < 8.3 pg/mL, for eotaxin < 4.0 pg/mL, for IP-10 < 2.6 pg/mL, and for MCP-1 < 1.9 pg/mL. The intra-assay percent coefficient of variation (%CV) was <10% and the interassay %CV < 20% for the analytes in both kits.

When concentrations of cytokines were in the lower range, the analysis software determined the concentration as “out of range.” For these samples, the level was considered as zero, since the true concentration cannot be calculated and can be any value between 0 and LOD. For each cytokine, the number of samples with zero levels were calculated. As a quality control, cytokines with >50% of samples “out of range” were excluded from further analysis. Samples before and after exercise in FM were analyzed in the same kit.

### 2.9. Statistics

#### 2.9.1. Univariate Analyses

Statistical analyses were made using Statistica version 12 (StatSoft Inc., Tulsa, OK, USA) and SigmaPlot version 13 (SysStat Inc., San José, CA, USA). Kolmogorov-Smirnov test was used to test for normality of data. None of the cytokines were normally distributed. An attempt was therefore made to logarithmic (ln) transform data, but data remained nonnormally distributed.

Descriptive data are presented as mean and standard deviation (SD) or median and interquartile range (IQR). Baseline data were compared between FM and controls with independent *t*-test, Mann–Whitney *U* test or chi^2^ test depending on the distribution of data.

For changes in data between interventions (exercise and relaxation), the absolute change (Δ) was calculated and used for comparison using independent *t*-test or Mann–Whitney *U* test.

#### 2.9.2. Multivariate Analyses

Data sets obtained from biochemical analyses, for example, from the “omics” field (e.g., proteomics) are generally characterized by a large number of intercorrelated molecules/proteins and low subject-to-variables ratios, so these methods often violate central assumptions of traditional statistical methods [35]. Thus, it is necessary to use modern multivariate data analysis methods (MVDA) such as advanced principal component analysis (PCA) and different types of partial least square discriminant analyses (PLS-DA) [35]. Hence, MVDA can be used to separate, quantify, and determine the important pattern of biochemical substances from plasma.

MVDA were performed using SIMCA v.13.0 (UMETRICS, Umeå, Sweden). When applying MVDA, the recommendations presented by Wheelock AM and Wheelock CE were essentially followed [35]. Variables were mean centered and scaled for unified variance (UV-scaling). An unsupervised PCA was first used to check multivariate outliers among the subjects with respect to cytokines [36]. PCA also enables the identification of multivariate outliers, as assessed by Hotelling's T2 statistic (T2 critical 95%, identifies strong outliers) and by distance to model in X-space (DModX, identifies moderate outliers—DModX twice as large as DCrit was applied as criterion for serious moderate outliers). The removal of strong outliers is crucial, as a few strong outliers may influence the model in a detrimental way; in this study, four strong outliers were identified (two of each group) and they were excluded from the subsequent multivariate analyses. PCA was also used for understanding the multivariate correlation pattern. Uncorrelated principal components (*p*) are obtained and the loading for each variable indicates the degree of correlation with the component. Variables with high absolute loadings (<0.20 as a rule of thumb) upon a principal component are intercorrelated; the same sign indicates a positive intercorrelation while different signs for two variables with high loadings indicate a negative correlation. In the next step, when investigating the multivariate correlations between the cytokines and group membership, orthogonal partial least squares discriminant analysis (OPLS-DA) was applied [36]. In the OPLS-DA, variables (regressors) were considered important if they had regression coefficients with a jack-knifed 95% confidence interval not including 0 and a variable of importance (VIP) value greater than 1. When appropriate the OPLS-DA analysis was made in two steps. First, all cytokines were included, and from this analysis, cytokines were selected with VIP > 1.0 combined with the jack-knifed confidence intervals in the coefficients plot not including zero and used in a new regression presented in the results. Coefficients (PLS scaled and centered regression coefficients) were used to note the direction of the relationship (positive or negative). *R* ^2^ describes the goodness of fit—the fraction of sum of squares of all the variables explained by a principal component. *Q* ^2^ describes the goodness of prediction—the fraction of the total variation of the variables that can be predicted by a principal component using cross validation methods. *R* ^2^ should not be considerably higher than *Q* ^2^. A difference greater than 0.2–0.3 implies overfitting, meaning that the robustness of the model is poor [36]. To validate the model, obtained cross-validated analysis of variance (CV-ANOVA) was used. The multivariate regressions were considered of significant importance if the CV-ANOVA had *P* < 0.05.

## 3. Results

The first comparison was made for baseline data between FM and controls. The sample consisted of the 125 FM women and 130 controls for which blood plasma was obtained at baseline.

### 3.1. Baseline Characteristics

Baseline characteristics of the participants are presented in Table 1. As can be seen, FM and controls differed in most anthropometric variables and functional as well as psychometric measures, although HADS depression and HADS anxiety scores were in the normal range (<10) in both groups.

### 3.2. Plasma Cytokine Levels at Baseline

Plasma cytokine levels in FM and controls are shown in Table 2.

IL-4 was only detectable in 44% of samples and was therefore excluded from further analysis. All other cytokines were detectable in between 70% (IL-1*β*) and 100% (IL-17A, TNF-*α*, IP-10, MCP-1, and eotaxin). There were fewer samples with detectable levels of IL-1*β* (chi^2^ test, *P* < 0.001) and IL-1ra (*P* = 0.026) in FM (55% and 83%), compared to controls (70% and 93%).

The levels of IL-2, IL-6, TNF-*α*, and eotaxin were higher in FM compared to the controls, while IL-1*β* was lower in FM.

### 3.3. Group Differences in the Multivariate Context

In order to confirm the traditional bivariate comparisons of the cytokines reported in Table 2, we in the next step analyzed which variables were important in the multivariate context with respect to group belonging using PLS-DA regression. Hence, in contrast to the traditional statistical tests reported above, the multivariate intercorrelation pattern between the cytokines was considered. IL-2 (VIP = 1.79), IL-6 (VIP = 1.60), IL-1*β* (VIP = 1.15), eotaxin (VIP = 1.13), and IL-17A (VIP = 1.10) were the most important cytokines (i.e., VIP > 1) that differentiated between the two groups of subjects (*R* ^2^ = 0.07, *Q* ^2^ = 0.02, CV-ANOVA *P* = 0.05); they were higher in FM except for IL-1*β* (lower).

### 3.4. Multivariate Correlation Pattern between Background Data, Psychometric Data, and Cytokines

In order to understand if significant relationships existed between the levels of cytokines and psychometric data a PCA was done (Table 3). Four components were obtained in the significant PCA. The first component (p1) mainly showed interrelationships between psychometric variables and physical performance. The second component (p2) was mainly determined by certain cytokines. The third component was dominated by a positive correlation pattern between age, blood pressures and eotaxin. The fourth component (p4) mainly reflected positive correlations between weight, BMI, and physical tests. Hence, the PCA did not indicate strong intercorrelations between cytokines and other variables. However, more in-depth analyses—reported in the subsequent paragraphs—revealed some significant relationships even though with low *R* ^2^.

### 3.5. The Relationships between Cytokines and Other Variables

#### 3.5.1. Regression of Pain Intensity, FIQ, and PCS in FM

No significant and/or stable models were achieved for VAS, FIQ, and PCS in FM using the cytokines as regressors.

#### 3.5.2. Regressions of PPT

In Table A (Supplementary material available here), regressions of PPT are shown. When all subjects were included (both FM and controls), a significant regression was obtained. This was also the case when the controls were analyzed separately. No significant regression was found in FM. The obtained models were significant per CV-ANOVA but low *R* ^2^ and *Q* ^2^ were found.

In both groups taken together, IL-10, IL-17A, IL-2, IL-6, TNF-*α*, and IFN-Y were the most important regressors and correlated negatively with PPT. Hence, high levels of these cytokines were associated with low PPT values.

In the controls, IL-6, IL-10, IL-17A, IFN-Y, and IL-1ra were the most important regressors (negatively correlated) of PPT.

#### 3.5.3. Regressions of HADS Depression, HADS Anxiety, and SF36-MCS

No significant regressions of HADS depression, HADS anxiety, or SF36-MCS were obtained for all subjects or the two groups separately using the cytokines as regressors.

#### 3.5.4. Regression of PDI in FM

Regressions of PDI in FM are shown in Table B (Supplementary material). A significant regression of PDI was obtained (*R* ^2^ = 0.19, *Q* ^2^ = 0.08, CV-ANOVA *P* = 0.05). According to the regression, IL-10 and IL-2 (both positively) and MCP-1, IL-1ra, eotaxin, and IP-10 (all negatively) were significant regressors of PDI in FM.

### 3.6. Change in Clinical and Functional Measures after Interventions

Table 4 summarizes the clinical and functional measures before and after the interventions for the participants in this substudy. For a full description of the results of the RCT, please see our main article [23]. The two FM groups did not differ in clinical and functional measures at baseline.

### 3.7. Change of Plasma Cytokines after Interventions

Table 5 shows the cytokine levels at baseline and after the interventions in the two groups. Blood samples obtained both before and after training were available from 92 women, 49 in the exercise group and 43 in the relaxation group.

At baseline, there were no significant differences between the exercise and relaxation groups in cytokine levels, except for IL-8 that was higher in the exercise group. IL-1ra had increased in both groups after the intervention (*P* = 0.004). IL-1*β* had increased in the relaxation group (*P* = 0.002) and ΔIL-1*β* was greater in the relaxation group than in the exercise group. There were no other group differences in cytokine levels.

### 3.8. The Changes of Cytokine Levels before and after the Intervention in the Multivariate Context

Since we found no differences in changes of cytokines between the two interventions in the univariate statistical analyses, the subsequent paragraphs concern the two intervention groups taken together. The multivariate correlation pattern according to PCA of between changes in cytokines identified one significant component (*R* ^2^ = 0.38; *Q* ^2^ = 0.28); that is, changes in IFN-*γ*, IL-2, IL-6, IL-8, IL-10, IL-17A, and TNF-*α* were positively intercorrelated (loadings: 0.33–0.40).

### 3.9. The Relationships between Changes in Cytokines and Other Variables

**Figure 2:**
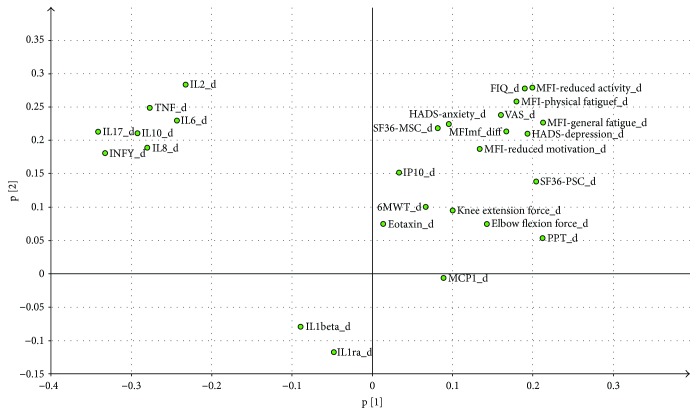
Loading plot (the relationships between variables) based on the changes (d) in cytokines and changes in clinical variables in FM. Two components were identified.

The loadings for the two components are shown in Figure 2 and Table C (Supplementary material). A PCA identified two significant components (*R* ^2^ (cumulative) = 0.35; *Q* ^2^ (cumulative) = 0.18) and the loading plot is shown in Figure 1. This analysis indicated that only weak correlations existed between the changes in most cytokines and changes in clinical variables such as VAS, MFI, PPT, and FIQ. Hence, generally no significant regressions could be established for these variables separately using the changes in cytokines as regressors except for changes in PPT.

#### 3.9.1. Regression of Changes in PPT Using Changes in Cytokines as Regressors

**Figure 3:**
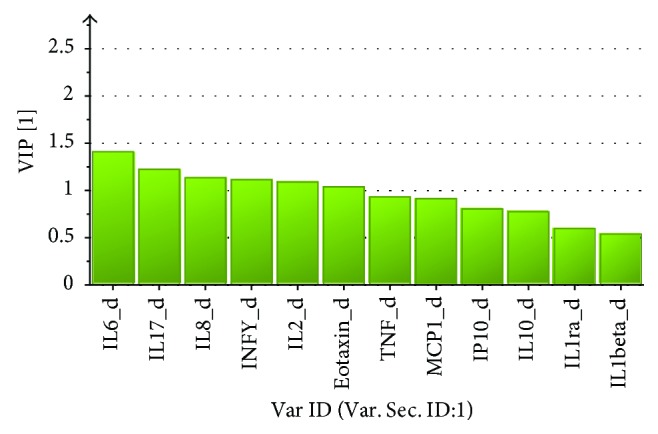
VIP values for the OPLS regression of changes in PPT using the changes in cytokines (diff) as regressors. VIP > 1.0 is significant.

Figure 3 shows the changes in PPT using changes in cytokines as regressors. A significant model was obtained (*R* ^2^ = 0.12, *Q* ^2^ = 0.07, CV-ANOVA *P* = 0.04). Hence, changes in IFN-*γ*, IL-2, IL-6, IL-8, and IL-17A were multivariately correlated with changes in PPT. However, even though the regression was significant, the explained variation in changes in PPT (*R* ^2^ = 0.12) was low.

#### 3.9.2. Are Levels of Cytokines at Baseline Indicative of Changes in Clinical Variables after the Interventions?

Table D (Supplementary material) shows correlations between changes of baseline levels of the cytokines and changes in the clinical variables. The PCA identified two significant components (*R* ^2^ (cumulative) = 0.32; *Q* ^2^ (cumulative) = 0.12). Hence, these results indicated weak correlations between cytokines at baseline and changes in clinical variables. It was not possible to regress changes in single clinical variables using cytokine levels at baseline.

## 4. Discussion

This is one of the larger studies performed investigating circulating cytokine levels in FM compared to age- and gender-matched healthy controls. We found that several cytokines and chemokines showed elevated plasma concentrations in FM. Even if not all cytokines differed between groups, in general, our first hypothesis was confirmed. On the contrary, the second hypothesis, that a progressive resistance exercise intervention would normalize circulating cytokine levels was not confirmed, only the anti-inflammatory IL-1ra had changed significantly, but independent of whether participants were in the intervention group or in the active control group. The results of the interventions on subjective symptoms as well as clinical and functional changes have been reported in our main article [23], which is why they are not discussed in this substudy.

### 4.1. Baseline Cytokine Levels

It has been suggested by several researchers that chronic low-grade systemic inflammation may underlie the pathophysiology in chronic generalized pain conditions, such as FM [14]. This study lended further support to this suggestion and was congruent with previous studies of elevated circulating cytokine levels in FM [11–13, 15]. Using multiplex panels analyzing 13 cytokines and chemokines, the univariate analyses showed increased levels of the proinflammatory IL-2, IL-6, TNF-*α*, IP-10, and eotaxin. The multivariate analysis—also considering the intercorrelation pattern between cytokines—revealed a slightly different pattern with IL-2, IL-6, IL-1*β*, eotaxin, and IL-17A being the most important cytokines that differentiated FM from controls.

Increased circulating levels of these cytokines have been reported previously in FM [13, 37–42]. However, many other studies have not found differences in cytokine levels between FM and controls, as shown in the systematic review with meta-analysis by Uceyler et al.; only for IL-6, IL-8, and IL-1ra was there evidence for elevated levels [11]. Although IL-8 did not differ significantly between groups in this study, the median level was 50% higher in FM. As several other studies have reported elevated circulating levels of IL-8 [11, 43], the lack of significance could perhaps be explained by a larger interindividual variation compared to other studies [44] or different study samples. For example, in our study, the participants were not patients in tertiary care, but FM women volunteering to participate in the RCT regarding progressive resistance training, which is why they could be expected to be healthier. This might also explain the lack of increased IL-1ra level compared to previous studies [45, 46]. While most previous studies have reported lack of differences in circulating IL-1*β* l [11], the lower levels of IL-1*β* in FM compared to controls in our study are in line with the results from a study by Kosek and coworkers [44].

We also found increased levels of TNF in the vastus lateralis muscle in a subgroup of women with FM from this study compared to controls [47]. In CSF, IL-8 [43, 44] has been reported to be elevated, and Bäckryd et al. [15] using multivariate analysis showed that several other cytokines and chemokines in CSF and plasma discriminated FM from controls. Further, mRNA expression of IL-10 in CSF was increased in FM [48]. On the contrary, Sturgill and coworkers [14] reported reduced plasma levels of the anti-inflammatory cytokines IL-4, IL-5, and IL-13 in FM. The results of that study are hampered by a small patient sample and that no control group was used, even if they were confirmed in an independent patient sample. However, Uceyler [49] also reported reduced levels of IL-4 and IL-10 in widespread pain (65% FM patients). This may be explained by an imbalance between Th1 (proinflammatory) and Th2 cytokines (anti-inflammatory) [14] or a ceiling effect evident in some patients. Together, these results show that several cytokines may differentiate FM from controls. However, given the low *R* ^2^ and *Q* ^2^ values, differences in cytokine pattern between FM and controls only had little influence on group belonging.

There were only weak relations between cytokines and clinical variables, although many of these models were highly significant according to the *P* values from CV-ANOVAs. This is in agreement with a study in widespread pain patients, in which 88% fulfilled the criteria for FM [50]. Previously, some other studies have reported correlations between IL-6 and IL-17A and pain and depression, but few of these have used multivariate analyses. Other studies have not found any correlations between L-6, IL-8, IL-10, TNF-*α*, FIQ and depression [51], IL-6 and depression or anxiety [52], IL-6 and pain score, FIQ, or TNF-*α* [53]. Collectively, this means that peripheral cytokines, although of some importance, probably have only little influence on the clinical phenotype of FM.

### 4.2. Cytokine Levels after Exercise

The results of the interventions on subjective symptoms as well as on clinical and functional data have been reported in our main article [23]. Briefly, they showed that the participants in the progressive resistance exercise group had increased their muscle strength in general [23]. Though in this subset of participants only isometric knee-extension force changed, neither the univariate nor the multivariate statistics could detect any significant changes in plasma cytokine levels after the exercise apart from an increase of IL-1ra. However, this increase was also found after relaxation, which is why it is unlikely that it is an effect of the exercise. It is well described that during exercise, IL-6 is produced in muscles via a TNF-independent pathway. This stimulates the release of anti-inflammatory cytokines, such as IL-1ra and IL-10, and inhibits the release of TNF-*α* [54]. In our previous microdialysis study, performed in a subgroup of the FM women from the multicenter study, muscle IL-6 had increased significantly directly after repetitive muscle contraction, in line with this [47, 55]. However, not only IL-6 is released in muscle tissue; up to 600 proteins have been reported to be secreted from muscle cells, although for most of them, their responses to exercise are not well-known [56]. Studies have also shown that short-duration exhausting exercise increase the release of circulating IL-1*β*, IL-4, IL-8, and MCP-1, in addition to IL-6 and IL-1ra [57, 58]. The type of exercise and the intensity seem to influence on the plasma cytokine release, such that more exhaustive exercise at higher intensity produces the highest levels [59]. In FM, a blunted release of circulating IL-10 was reported following exhaustive exercise supporting reduced anti-inflammatory response to exercise [24]. It should also be noted that there is a discrepancy between the levels of some cytokines in muscle tissue and in plasma following exercise. Some cytokines that are secreted in the muscle do not seem to enter the circulation or are not secreted in sufficient quantities to reach the circulation, while for other cytokines, skeletal muscle is probably not the major source [58].

The positive effects of regular physical activity of moderate intensity on the immune system have been discussed before [60]. In individuals with systemic low-grade inflammation, for example the obese, the effect seems to be greater [60]. Also in FM, studies have reported positive effects of regular exercise on inflammatory markers [26, 61]. While most previous studies have dealt with aerobic exercise, less is known about the effect of resistance exercise on the immune system. Previous data show that long-term resistance training results in lower basal levels of some proinflammatory cytokines, but that only high-intensity resistance exercise increase the production of anti-inflammatory cytokines [62]. Although IL-1ra had increased after both interventions in this study, the mechanisms behind its increase could differ. Therefore, the increase of IL-1ra after the interventions could perhaps indicate that the resistance exercise was of high enough intensity to increase its production. Results from two large randomized aerobic exercise intervention trials suggest that aerobic exercise does not alter IL-10 or IL-4 [63], which if so may explain why IL-1ra was the only anti-inflammatory cytokine that had increased. However, there were no changes in proinflammatory cytokines. Perhaps our participants were healthier than in other studies of FM (as discussed regarding IL-8 above), since the effect of exercise on circulating cytokine levels in previously sedentary, but otherwise healthy, individuals seem to be only modest [64]. Hence, the positive effects on muscle strength found in our RCT was most probably not mediated by changes in the inflammatory response. This is in line with the results from our microdialysis study, which neither showed any significant changes of muscle cytokines after the interventions [55]. In contrast, increased levels of glutamate and pyruvate before the exercise were normalized after exercise [65], why the effect may instead be due to metabolic alterations.

Previous studies have shown a beneficial effect of relaxation on the immune system. Studies have shown that relaxation can modulate the immune response, for example, by increasing the number of CD4-positive T cells [66]. These cells are a source of IL-4 that promotes the production of Th2 cytokines and they stimulate the production of IL-1ra from stimulated monocytes and hepatocytes [67]. One may speculate that this perhaps could be a possible mechanism to the increase of IL-1ra in the active control group.

There were only weak correlations between changes in cytokine levels and VAS, MFI, PPT, and FIQ (Table C; Additional File 1), but it was not possible to regress these clinical variables. Neither was it possible to regress changes in HADS using changes in cytokines. This contrasts with a recent study that showed associations between changes of IL-6 and depressive symptoms after exercise, thereby providing support for a plausible involvement of IL-6 in the antidepressive effect of exercise [68]. On the other hand, the HAD scores were within the normal range in the FM groups which may explain the different results. However, changes in PPT could be significantly regressed using changes in IFN-*γ*, IL-2, IL-4, IL-6, IL-8, and IL-17A, but the correlations were weak and changes in cytokine levels only explained a minor part of changes in PPT. This is in concordance with a study in FM subjected to 15 weeks of Nordic walking or low-intensity walking, where changes in IGF-1 correlated with changes in PPT [27]. Neither did baseline levels of cytokines have a major impact on changes in clinical variables after the interventions.

We did not control for the circadian release of cytokines. Studies have shown higher levels of several cytokines in the afternoon compared to in the morning [69]. This may be regarded as a limitation. However, in both patients and controls, blood was drawn during the same time span, meaning that the diurnal variation would have been leveled out between participants in both groups. We therefore consider this of minor importance for the results.

Another study limitation is that we did not match participants according to BMI; the FM group had higher BMI and higher blood pressure than the healthy baseline controls and we cannot exclude that some of the participants had concomitant metabolic syndrome. Studies have shown that there is a link between metabolic syndrome and circulating cytokine levels [70], which is why this may have affected our results.

## 5. Conclusion

Within the limitations of this study, we conclude that in line with previous reports, plasma levels of several cytokines seem to be elevated in FM. This supports the hypothesis that chronic systemic inflammation may underlie its pathophysiology. However, the relation to clinical variables was weak. Although progressive resistance exercise and relaxation significantly increased IL-1ra levels, no other cytokines changed and the correlations between changes in cytokine levels and in clinical variables were weak. This suggests that 15 weeks of resistance exercise, as performed in this study, did not show any anti-inflammatory effect on neither FM symptoms nor clinical and functional variables.

## Figures and Tables

**Table 1:** Background data of women with fibromyalgia (FM) and healthy controls (HC).

Variables	FM *n* = 125	HC *n* = 130	*P* value
Age (yr)	51.2 (±9.4)	48.2 (±11.4)	0.124
BMI (kg/m^2^)	28.0 (±5.2)	24.2 (±3.8)	*<0.001*
Systolic BP (mmHg)	130.0 (±18.9)	123.3 (13.0)	*0.001*
Diastolic BP (mmHg)	81.6 (±9.1)	77.8 (±8.8)	*0.002*
Medication (%)			
Antidepressants (SSRI, SNRI, TCA)	43.9	0	***—***
Anticonvulsants	4.9	0	***—***
Pain duration (yr)	10.5 (±7.7)	NA	**—**
Tender points (*n*)	15.7 (±1.9)	NA	***—***
VAS global pain (0–100)	52.0 (34.0)	0 (0)	*<0.001*
PPT (kPa)	184.8 (±80.1)	357.9 (±108.9)	*0.001*
Hand grip force (N)	192.3 (±73.8)	275.0 (±50.8)	*<0.001*
Elbow flexion force (kg)	12.0 (5.4)	18.2 (5.2)	*<0.001*
Knee extension force (N)	315.4 (±110.4)	411.0 (88.3)	*<0.001*
6MWT (m)	551.4 (±71.0)	656.9 (±60.1)	*<0.001*
PDI (0–70)	37.0 (17.0)	7.0 (0.0)	*<0.001*
HADS depression (0–21)	7.0 (4.0)	1.0 (3.0)	*<0.001*
HADS anxiety (0–21)	8.0 (6.0)	3.0 (4.0)	*<0.001*
PCS (0–52)	20.0 (17.0)	5.0 (12.0)	*<0.001*
SF36-PSC (0–100)	30.3 (9.8)	56.4 (3.8)	*<0.001*
SF36-MSC (0–100)	38.5 (19.0)	57.7 (5.7)	*<0.001*
FIQ (0–100)	61.1 (24.9)	3.5 (9.1)	*<0.001*
MFI (0–20)			
General fatigue	19.0 (4.0)	7.0 (4.0)	*<0.001*
Physical fatigue	17.0 (5.0)	5.0 (5.0)	*<0.001*
Reduced activity	15.0 (5.0)	6.0 (4.5)	*<0.001*
Reduced motivation	10.0 (5.0)	5.0 (2.5)	*<0.001*
Mental fatigue	16.0 (5.0)	7.0 (4.0)	*<0.001*

Data are presented as mean (±SD) or median (interquartile range). BMI = body mass index; BP = blood pressure; FIQ = fibromyalgia impact questionnaire; HADS = hospital anxiety and depression scale; MFI = multidimensional fatigue inventory; PDI = pain disability index; PPT = pressure pain thresholds (mean of 8 sites); SF36 = Short Form Health Survey 36, physical (PSC) and mental summary components (MSC); SNRI = serotonin and noradrenaline reuptake inhibitor; SSRI = selective serotonin reuptake inhibitor; TAC = tricyclic antidepressant; 6MWT = 6-min walk test; VAS = visual analogue scale. Italic texts denote significant differences (*t*-test or Mann–Whitney *U* test; *P* < 0.05).

**Table 2:** Cytokine levels at baseline in plasma from women with fibromyalgia (FM) and healthy controls (HC).

Cytokine	FM *n* = 125	HC *n* = 130	*P*-value
IFN-*γ*	9.9 (9.3)	9.3 (10.1)	0.408
IL-1*β*	0.8 (1.8)	1.4 (2.0)	*0.001*
IL-2	2.9 (3.9)	2.4 (2.4)	*0.041*
IL-6	2.0 (2.3)	1.6 (1.5)	*0.009*
IL-8	1.4 (2.2)	0.9 (1.9)	0.150
IL-10	7.9 (8.0)	7.6 (7.2)	0.415
IL-17A	9.8 (8.6)	7.9 (7.8)	0.151
TNF-*α*	4.3 (2.1)	3.6 (2.6)	*0.013*
IL-1ra	33.0 (116.0)	41.5 (74.9)	0.372
IP-10	398.5 (229.9)	346.4 (254.4)	*0.013*
MCP-1	310.3 (136.8)	298.7 (115.4)	0.627
Eotaxin	107.5 (50.1)	97.9 (40.9)	*0.024*

Data are presented as median (interquartile range). Italic figures denote significant differences (Mann–Whitney *U* test; *P* < 0.05).

**Table 3:** PCAs of background data, psychometric, and cytokines in fibromyalgia and healthy controls taken together.

Variables	p[1]	p[2]	p[3]	p[4]
Age	0.06	−0.14	**0.34**	−0.15
Weight	0.12	−0.06	0.17	**0.41**
Height	−0.02	−0.19	−0.06	0.19
BMI	0.15	−0.07	**0.20**	**0.36**
Systolic BP	0.07	−0.19	**0.34**	0.13
Diastolic BP	0.08	−0.13	**0.34**	0.16
VAS global pain intensity	**0.29**	−0.03	−0.13	0.03
PPT	**−0.23**	−0.04	0.07	−0.04
Hand grip force	**−0.25**	−0.03	−0.12	**0.33**
Elbow flexion force	**−0.26**	−0.04	−0.13	**0.32**
Knee extension force	**−0.21**	−0.02	−0.13	**0.40**
6MWT	**−0.26**	0.12	−0.15	0.02
PDI	**0.20**	−0.07	−0.20	0.02
HADS depression	**−0.22**	−0.11	**0.23**	−0.11
HADS anxiety	**0.25**	−0.07	**−0.22**	0.10
PCS	**0.30**	−0.08	−0.13	0.09
SF36-PCS	**0.30**	−0.07	−0.13	0.06
SF36-MCS	**−0.29**	0.07	0.05	−0.06
FIQ	**−0.24**	−0.11	−0.15	**0.27**
IFN-*γ*	0.08	**0.34**	0.11	0.06
IL-1*β*	**0.21**	−0.08	**−0.23**	0.09
IL-2	−0.04	**0.20**	−0.01	0.03
IL-6	0.06	**0.39**	0.00	0.13
IL-8	0.00	0.07	0.03	0.11
IL-10	0.09	**0.27**	0.06	0.07
IL-17A	0.10	**0.35**	−0.02	0.13
TNF-*α*	0.07	**0.40**	0.02	0.10
IL-1ra	0.08	**0.36**	0.08	0.05
IP-10	0.03	−0.03	0.11	0.06
MCP-1	0.04	0.07	**0.21**	0.18
eotaxin	0.01	−0.08	**0.30**	0.08

*R* ^2^	0.26	0.11	0.09	0.07
*Q* ^2^	0.23	0.08	0.03	0.04

PCA = principle component analysis. Four significant components (p[1]-p [4]) were obtained. For each component are reported the loadings of the included variables. High absolute loadings (>0.20) are most important for the component. Variables with high absolute loadings are intercorrelated; the same sign indicate a positive intercorrelation while different signs for two variables with high loadings indicate a negative correlation. *R* ^2^ and *Q* ^2^ are reported for each component at the two bottom rows. *R* ^2^ (cumulative) = 0.53, *Q* ^2^ (cumulative) = 0.34. The loadings of the most important variables for the four components are denoted with bold type. BMI = body mass index; BP = blood pressure9; FIQ = fibromyalgia impact questionnaire; HADS = hospital anxiety and depression scale; PDI = pain disability index; PPT = pressure pain thresholds (mean of 8 sites); SF36 = Short Form Health Survey 36, physical (PSC) and mental summary components (MSC); 6MWT = 6-min walk test; VAS = visual analogue scale.

**Table 4:** Change in clinical and functional measures after interventions in women with fibromyalgia.

	Exercise		Relaxation	
	*n* = 49		*n* = 43	
	Before	Δ	Before	Δ
VAS global pain intensity (0–100)	50.0 (36.0)	−16.0 (40.0)	56.0 (26.0)	−*2.0 (18.0)*
PPT (kPa)	178.6 (±77.1)	7.3 (±67.0)	183.1 (±88.7)	−15.9(±46.9)
Hand grip force (N)	196.2 (±70.9)	21.0 (±40.0)	177.6 (±72.5)	20.3 (±46.0)
Elbow flexion force (kg)	12.4 (±4.9)	2.1 (±3.1)	11.1 (±5.3)	1.3 (±3.6)
Knee extension force (N)	321.6 (±108.2)	29.3(±69.3)	295.3 (±111.9)	*−3.0 (±68.6)*
6MWT (m)	565.4 (±75.4)	17.3 (±63.1)	542.0 (±67.4)	*−4.6 (±44.2)*
PDI (0–70)	38.0 (17.0)	−5.0 (13.0)	36.0 (15.5)	*0.0 (11.0)*
HADS-depression (0–21)	7.0 (5.0)	−1.0 (4.0)	7.0 (4.0)	*0.0 (2.0)*
HADS-anxiety (0–21)	8.0 (6.0)	0.0 (5.0)	8.0 (6.0)	*0.0 (3.0)*
PCS (0–52)	18.0 (16.0)	−3.0 (11.0)	19.0 (18.5)	*−2.0 (8.5)*
SF36-PSC (0–100)	31.2 (9.4)	1.7 (11.7)	28.5 (10.4)	*1.3 (6.9)*
SF36-MSC (0–100)	36.6 (16.6)	2.5 (8.6)	41.4 (20.2)	*0.7 (12.0)*
FIQ (0–100)	57.1 (23.4)	−7.1 (15.5)	60.9 (26.0)	*−0.6 (19.9)*
MFI (0–20)				
General fatigue	19.0 (4.0)	−1.0 (3.0)	19.0 (3.5)	0.0 (1.8)
Physical fatigue	16.0 (4.0)	−2.0 (5.3)	17.0 (5.0)	*0.0 (2.0)*
Reduced activity	14.0 (6.0)	−1.0 (4.3)	15.0 (5.5)	0.0 (3.0)
Reduced motivation	10.0 (5.0)	−1.0 (4.0)	9.0 (5.0)	0.0 (3.0)
Mental fatigue	16.0 (5.0)	−2.0 (4.3)	16.0 (6.0)	*0.0 (3.8)*

Data are presented as mean (±SD) or median (interquartile range) before and their change (Δ) after 15 weeks of progressive resistance exercise or relaxation therapy. Note that these data essentially have been published elsewhere [23]. BMI = body mass index; BP = blood pressure; FIQ = fibromyalgia impact questionnaire; HADS = hospital anxiety and depression scale; MFI = multidimensional fatigue inventory; PDI = pain disability index; PPT = pressure pain thresholds (mean of 8 sites); SF36 = Short Form Health Survey 36, physical (PSC) and mental summary components (MSC); 6MWT = 6-min walk test; VAS = visual analogue scale. Italic figures denote significant group difference (independent *t*-test/Mann–Whitney *U* test; *P* < 0.05).

**Table 5:** Changes in cytokine levels before and after the interventions in women with fibromyalgia.

pg/mL	Exercise		Relaxation	
	*n* = 49		*n* = 43	
	Before	Δ	Before	Δ
IFN-*γ*	11.2 (9.0)	−1.8 (9.2)	9.7 (12.3)	−0.4 (8.6)
IL-1*β*	0.9 (1.9)	0.0 (1.3)	0.0 (1.6)	*0.6 (1.6)*
IL-2	3.3 (4.9)	−0.3 (2.7)	2.8 (3.1)	−0.3 (1.6)
IL-6	1.7 (2.5)	−0.2 (1.2)	2.1 (2.4)	0.2 (1.9)
IL-8	1.6 (1.9)#	−0.3 (1.6)	0.8 (1.7)	0.0 (1.3)
IL-10	8.0 (9.7)	−1.0 (6.3)	7.9 (9.6)	0.8 (7.4)
IL-17A	10.8 (10.1)	−2.1 (7.6)	9.2 (7.5)	−0.7 (5.9)
TNF-*α*	4.5 (2.2)	−0.4 (1.8)	4.0 (1.7)	−0.3 (2.0)
IL-1ra	27.9 (67.8)	22.1 (60.8)	44.7 (105.4)	21.4 (91.5)
IP-10	379.7 (230.4)	2.3 (180.4)	398.5 (219.7)	−33.5 (184.3)
MCP-1	318.0 (124.5)	7.1 (131.5)	289.6 (151.0)	23.6 (76.3)
eotaxin	107.0 (42.6)	−11.4 (53.0)	106.9 (52.6)	−8.6 (34.6)

Data show median (interquartile range) cytokine levels before and their change (Δ) after 15 weeks of progressive resistance exercise or relaxation therapy. Italic font indicates significant difference between groups (Mann–Whitney U-test; *P* = 0.037).

## Data Availability

As more analyses from this RCT are currently underway, the authors will not share their data until all analyses have been made.

## Supplementary Materials

Supplementary Materials Table A: the table shows OPLS regression of mean PPT using cytokines as regressors in all subjects taken together and in CON. VIP > 1.0 is significant. The sign of CoeffCS and p(corr) shows the direction of the correlation. Thus, negative values indicate that this variable is low in FMS and a positive value that this variable is high in FMS. The three bottom rows report *R* ^2^, *Q* ^2^, and CV-ANOVA (*P* value). Table B: OPLS regression of PDI in FMS using cytokines as regressors. VIP > 1.0 is significant. The sign of CoeffCS and p(corr) shows the direction of the correlation. Thus, negative values indicate that this variable is low in FMS and a positive value that this variable is high in FMS. The three bottom rows report *R* ^2^, *Q* ^2^, and CV-ANOVA (*P* value). Table C: PCAs of changes in cytokines and clinical psychometric instruments in both intervention groups taken together. Two significant components (p[1] and p[2]) were obtained. For each component are reported the loadings of the variables. High absolute loadings are most important for the component. Variables with high absolute loadings are intercorrelated; the same sign indicate a positive intercorrelation while different signs for two variables with high loadings indicate a negative correlation. *R* ^2^ and *Q* ^2^ are reported for each significant component at the two bottom rows. The loadings of the most important variables for the two components are denoted with bold type. See also Figure 1. Table D: PCAs of baseline cytokine levels and changes in psychometric instruments in both groups taken together. Two significant components (p[1] and p[2]) were obtained. For each component are reported the loadings of the variables. High absolute loadings are most important for the component. Variables with high absolute loadings are intercorrelated; the same sign indicate a positive intercorrelation while different signs for two variables with high loadings indicate a negative correlation. *R* ^2^ and *Q* ^2^ are reported for each significant component at the two bottom rows. The loadings of the most important variables for the two components are denoted with bold type.

## References

[1] Clauw D. J. (2015). Fibromyalgia and related conditions. *Mayo Clinic Proceedings*.

[2] McNally J. D., Matheson D. A., Bakowsky V. S. (2006). The epidemiology of self-reported fibromyalgia in Canada. *Chronic Diseases in Canada*.

[3] Wolfe F., Smythe H. A., Yunus M. B. (1990). The American College of Rheumatology 1990 criteria for the classification of fibromyalgia. *Arthritis and Rheumatism*.

[4] Wolfe F., Clauw D. J., Fitzcharles M. A. (2010). The American College of Rheumatology preliminary diagnostic criteria for fibromyalgia and measurement of symptom severity. *Arthritis Care & Research*.

[5] Wolfe F., Clauw D. J., Fitzcharles M. A. (2016). 2016 revisions to the 2010/2011 fibromyalgia diagnostic criteria. *Seminars in Arthritis and Rheumatism*.

[6] Sluka K. A., Clauw D. J. (2016). Neurobiology of fibromyalgia and chronic widespread pain. *Neuroscience*.

[7] Cohen H., Neumann L., Kotler M., Buskila D. (2001). Autonomic nervous system derangement in fibromyalgia syndrome and related disorders. *The Israel Medical Association Journal*.

[8] Cunha F. Q., Lorenzetti B. B., Poole S., Ferreira S. H. (1991). Interleukin-8 as a mediator of sympathetic pain. *British Journal of Pharmacology*.

[9] Bote M. E., Garcia J. J., Hinchado M. D., Ortega E. (2012). Inflammatory/stress feedback dysregulation in women with fibromyalgia. *Neuroimmunomodulation*.

[10] Staud R. (2015). Cytokine and immune system abnormalities in fibromyalgia and other central sensitivity syndromes. *Current Rheumatology Reviews*.

[11] Uceyler N., Hauser W., Sommer C. (2011). Systematic review with meta-analysis: cytokines in fibromyalgia syndrome. *BMC Musculoskeletal Disorders*.

[12] Ang D. C., Moore M. N., Hilligoss J., Tabbey R. (2011). MCP-1 and IL-8 as pain biomarkers in fibromyalgia: a pilot study. *Pain Medicine*.

[13] Pernambuco A. P., Schetino L. P., Alvim C. C. (2013). Increased levels of IL-17A in patients with fibromyalgia. *Clinical and Experimental Rheumatology*.

[14] Sturgill J., McGee E., Menzies V. (2014). Unique cytokine signature in the plasma of patients with fibromyalgia. *Journal of Immunology Research*.

[15] Bäckryd E., Tanum L., Lind A. L., Larsson A., Gordh T. (2017). Evidence of both systemic inflammation and neuroinflammation in fibromyalgia patients, as assessed by a multiplex protein panel applied to the cerebrospinal fluid and to plasma. *Journal of Pain Research*.

[16] Ortega E., Bote M. E., Giraldo E., Garcia J. J. (2012). Aquatic exercise improves the monocyte pro- and anti-inflammatory cytokine production balance in fibromyalgia patients. *Scandinavian Journal of Medicine & Science in Sports*.

[17] Behm F. G., Gavin I. M., Karpenko O. (2012). Unique immunologic patterns in fibromyalgia. *BMC Clinical Pathology*.

[18] Wallace D. J., Gavin I. M., Karpenko O., Barkhordar F., Gillis B. S. (2015). Cytokine and chemokine profiles in fibromyalgia, rheumatoid arthritis and systemic lupus erythematosus: a potentially useful tool in differential diagnosis. *Rheumatology International*.

[19] Ericsson A., Mannerkorpi K. (2016). How to manage fatigue in fibromyalgia: nonpharmacological options. *Pain Management*.

[20] Mannerkorpi K., Iversen M. D. (2003). Physical exercise in fibromyalgia and related syndromes. *Best Practice & Research Clinical Rheumatology*.

[21] Garcia-Hermoso A., Saavedra J. M., Escalante Y. (2015). Effects of exercise on functional aerobic capacity in adults with fibromyalgia syndrome: a systematic review of randomized controlled trials. *Journal of Back and Musculoskeletal Rehabilitation*.

[22] Bidonde J., Busch A. J., Bath B., Milosavljevic S. (2014). Exercise for adults with fibromyalgia: an umbrella systematic review with synthesis of best evidence. *Current Rheumatology Reviews*.

[23] Larsson A., Palstam A., Lofgren M. (2015). Resistance exercise improves muscle strength, health status and pain intensity in fibromyalgia-a randomized controlled trial. *Arthritis Research & Therapy*.

[24] Torgrimson-Ojerio B., Ross R. L., Dieckmann N. F. (2014). Preliminary evidence of a blunted anti-inflammatory response to exhaustive exercise in fibromyalgia. *Journal of Neuroimmunology*.

[25] Gerdle B., Ghafouri B., Ernberg M., Larsson B. (2014). Chronic musculoskeletal pain: review of mechanisms and biochemical biomarkers as assessed by the microdialysis technique. *Journal of Pain Research*.

[26] Sanada K., Diez M. A., Valero M. S. (2015). Effects of non-pharmacological interventions on inflammatory biomarker expression in patients with fibromyalgia: a systematic review. *Arthritis Research & Therapy*.

[27] Bjersing J. L., Dehlin M., Erlandsson M., Bokarewa M. I., Mannerkorpi K. (2012). Changes in pain and insulin-like growth factor 1 in fibromyalgia during exercise: the involvement of cerebrospinal inflammatory factors and neuropeptides. *Arthritis Research & Therapy*.

[28] Tait R. C., Chibnall J. T., Krause S. (1990). The pain disability index: psychometric properties. *Pain*.

[29] Hedin P. J., Hamne M., Burckhardt C. S., Engstrom-Laurent A. (1995). The fibromyalgia impact questionnaire, a Swedish translation of a new tool for evaluation of the fibromyalgia patient. *Scandinavian Journal of Rheumatology*.

[30] Furst C. J., Ahsberg E. (2001). Dimensions of fatigue during radiotherapy. An application of the multidimensional fatigue inventory. *Support Care Cancer*.

[31] Sullivan M., Karlsson J. (1998). The Swedish SF-36 health survey III. Evaluation of criterion-based validity: results from normative population. *Journal of Clinical Epidemiology*.

[32] Petzke F., Khine A., Williams D., Groner K., Clauw D. J., Gracely R. H. (2001). Dolorimetry performed at 3 paired tender points highly predicts overall tenderness. *The Journal of Rheumatology*.

[33] Valkeinen H., Hakkinen A., Hannonen P., Hakkinen K., Alen M. (2006). Acute heavy-resistance exercise-induced pain and neuromuscular fatigue in elderly women with fibromyalgia and in healthy controls: effects of strength training. *Arthritis & Rheumatology*.

[34] Carville S. F., Arendt-Nielsen L., Bliddal H. (2008). EULAR evidence-based recommendations for the management of fibromyalgia syndrome. *Annals of the Rheumatic Diseases*.

[35] Wheelock A. M., Wheelock C. E. (2013). Trials and tribulations of ‘omics data analysis: assessing quality of SIMCA-based multivariate models using examples from pulmonary medicine. *Molecular BioSystems*.

[36] Eriksson L., Byrne T., Johansson E., Trygg J., Vikström C. (2013). *Multi- and Megavariate Data Analysis: Basic Principles and Applications*.

[37] Hernandez M. E., Becerril E., Perez M. (2010). Proinflammatory cytokine levels in fibromyalgia patients are independent of body mass index. *BMC Research Notes*.

[38] Gur A., Karakoc M., Nas K. (2002). Cytokines and depression in cases with fibromyalgia. *The Journal of Rheumatology*.

[39] Ghizal F., Das S. K., Verma N., Mahdi A. A. (2016). Evaluating relationship in cytokines level, fibromyalgia impact questionnaire and body mass index in women with fibromyalgia syndrome. *Journal of Back and Musculoskeletal Rehabilitation*.

[40] Zhang Z., Cherryholmes G., Mao A. (2008). High plasma levels of MCP-1 and eotaxin provide evidence for an immunological basis of fibromyalgia. *Experimental Biology and Medicine*.

[41] Garcia J. J., Cidoncha A., Bote M. E., Hinchado M. D., Ortega E. (2014). Altered profile of chemokines in fibromyalgia patients. *Annals of Clinical Biochemistry*.

[42] Feng J., Zhang Z., Wu X. (2013). Discovery of potential new gene variants and inflammatory cytokine associations with fibromyalgia syndrome by whole exome sequencing. *PLoS One*.

[43] Kadetoff D., Lampa J., Westman M., Andersson M., Kosek E. (2012). Evidence of central inflammation in fibromyalgia-increased cerebrospinal fluid interleukin-8 levels. *Journal of Neuroimmunology*.

[44] Kosek E., Altawil R., Kadetoff D. (2015). Evidence of different mediators of central inflammation in dysfunctional and inflammatory pain--interleukin-8 in fibromyalgia and interleukin-1*β* in rheumatoid arthritis. *Journal of Neuroimmunology*.

[45] Wallace D. J., Linker-Israeli M., Hallegua D., Silverman S., Silver D., Weisman M. H. (2001). Cytokines play an aetiopathogenetic role in fibromyalgia: a hypothesis and pilot study. *Rheumatology*.

[46] Iannuccelli C., Di Franco M., Alessandri C. (2010). Pathophysiology of fibromyalgia: a comparison with the tension-type headache, a localized pain syndrome. *Annals of the New York Academy of Sciences*.

[47] Christidis N., Ghafouri B., Larsson A. (2015). Comparison of the levels of pro-inflammatory cytokines released in the vastus lateralis muscle of patients with fibromyalgia and healthy controls during contractions of the quadriceps muscle--a microdialysis study. *PLoS One*.

[48] Light A. R., Bateman L., Jo D. (2012). Gene expression alterations at baseline and following moderate exercise in patients with chronic fatigue syndrome and fibromyalgia syndrome. *Journal of Internal Medicine*.

[49] Uceyler N., Valenza R., Stock M., Schedel R., Sprotte G., Sommer C. (2006). Reduced levels of antiinflammatory cytokines in patients with chronic widespread pain. *Arthritis & Rheumatology*.

[50] Gerdle B., Ghafouri B., Ghafouri N., Backryd E., Gordh T. (2017). Signs of ongoing inflammation in female patients with chronic widespread pain: a multivariate, explorative, cross-sectional study of blood samples. *Medicine*.

[51] Ranzolin A., Duarte A. L., Bredemeier M. (2016). Evaluation of cytokines, oxidative stress markers and brain-derived neurotrophic factor in patients with fibromyalgia - a controlled cross-sectional study. *Cytokine*.

[52] Nugraha B., Korallus C., Gutenbrunner C. (2013). Serum level of brain-derived neurotrophic factor in fibromyalgia syndrome correlates with depression but not anxiety. *Neurochemistry International*.

[53] Bazzichi L., Rossi A., Massimetti G. (2007). Cytokine patterns in fibromyalgia and their correlation with clinical manifestations. *Clinical and Experimental Rheumatology*.

[54] Pedersen B. K., Fischer C. P. (2007). Beneficial health effects of exercise--the role of IL-6 as a myokine. *Trends in Pharmacological Sciences*.

[55] Ernberg M., Christidis N., Ghafouri B. (2016). Effects of 15 weeks of resistance exercise on pro-inflammatory cytokine levels in the vastus lateralis muscle of patients with fibromyalgia. *Arthritis Research & Therapy*.

[56] Pedersen B. K. (2013). Muscle as a secretory organ. *Comprehensive Physiology*.

[57] Suzuki K., Nakaji S., Yamada M., Totsuka M., Sato K., Sugawara K. (2002). Systemic inflammatory response to exhaustive exercise. Cytokine kinetics. *Exercise Immunology Review*.

[58] Peake J. M., Della Gatta P., Suzuki K., Nieman D. C. (2015). Cytokine expression and secretion by skeletal muscle cells: regulatory mechanisms and exercise effects. *Exercise Immunology Review*.

[59] Nieman D. C., Konrad M., Henson D. A., Kennerly K., Shanely R. A., Wallner-Liebmann S. J. (2012). Variance in the acute inflammatory response to prolonged cycling is linked to exercise intensity. *Journal of Interferon & Cytokine Research*.

[60] Simpson R. J., Kunz H., Agha N., Graff R. (2015). Exercise and the regulation of immune functions. *Progress in Molecular Biology and Translational Science*.

[61] Ortega E., Garcia J. J., Bote M. E. (2009). Exercise in fibromyalgia and related inflammatory disorders: known effects and unknown chances. *Exercise Immunology Review*.

[62] Forti L. N., Van Roie E., Njemini R. (2017). Effects of resistance training at different loads on inflammatory markers in young adults. *European Journal of Applied Physiology*.

[63] Conroy S. M., Courneya K. S., Brenner D. R. (2016). Impact of aerobic exercise on levels of IL-4 and IL-10: results from two randomized intervention trials. *Cancer Medicine*.

[64] Simpson R. J., Lowder T. W., Spielmann G., Bigley A. B., LaVoy E. C., Kunz H. (2012). Exercise and the aging immune system. *Ageing Research Reviews*.

[65] Gerdle B., Ernberg M., Mannerkorpi K. (2016). Increased interstitial concentrations of glutamate and pyruvate in vastus lateralis of women with fibromyalgia syndrome are normalized after an exercise intervention - a case-control study. *PLoS One*.

[66] Brod S., Rattazzi L., Piras G., D'Acquisto F. (2014). ‘As above, so below’ examining the interplay between emotion and the immune system. *Immunology*.

[67] Gabay C., Porter B., Guenette D., Billir B., Arend W. P. (1999). Interleukin-4 (IL-4) and IL-13 enhance the effect of IL-1beta on production of IL-1 receptor antagonist by human primary hepatocytes and hepatoma HepG2 cells: differential effect on C-reactive protein production. *Blood*.

[68] Lavebratt C., Herring M. P., Liu J. J. (2017). Interleukin-6 and depressive symptom severity in response to physical exercise. *Psychiatry Research*.

[69] Altara R., Manca M., Hermans K. C. (2015). Diurnal rhythms of serum and plasma cytokine profiles in healthy elderly individuals assessed using membrane based multiplexed immunoassay. *Journal of Translational Medicine*.

[70] Grandl G., Wolfrum C. (2017). Hemostasis, endothelial stress, inflammation, and the metabolic syndrome. *Seminars in Immunopathology*.

