# Supplementary material for: Plasma Cytokine Levels in Fibromyalgia and Their Response to 15 Weeks of Progressive Resistance Exercise or Relaxation Therapy
Source: Mediators Inflamm. 2018 Apr 18;2018:3985154. doi: 10.1155/2018/3985154 (PMC5932448; doi:10.1155/2018/3985154)
Supplement: Supplementary Materials — Table A: the table shows OPLS regression of mean PPT using cytokines as regressors in all subjects taken together and in CON. VIP > 1.0 is significant. The sign of CoeffCS and p(corr) shows the direction of the correlation. Thus, negative values indicate that this variable is low in FMS and a positive value that this variable is high in FMS. The three bottom rows report R 2, Q 2, and CV-ANOVA (P value). Table B: OPLS regression of PDI in FMS using cytokines as regressors. VIP > 1.0 is significant. The sign of CoeffCS and p(corr) shows the direction of the correlation. Thus, negative values indicate that this variable is low in FMS and a positive value that this variable is high in FMS. The three bottom rows report R 2, Q 2, and CV-ANOVA (P value). Table C: PCAs of changes in cytokines and clinical psychometric instruments in both intervention groups taken together. Two significant components (p[1] and p[2]) were obtained. For each component are reported the loadings of the variables. High absolute loadings are most important for the component. Variables with high absolute loadings are intercorrelated; the same sign indicate a positive intercorrelation while different signs for two variables with high loadings indicate a negative correlation. R 2 and Q 2 are reported for each significant component at the two bottom rows. The loadings of the most important variables for the two components are denoted with bold type. See also Figure 1. Table D: PCAs of baseline cytokine levels and changes in psychometric instruments in both groups taken together. Two significant components (p[1] and p[2]) were obtained. For each component are reported the loadings of the variables. High absolute loadings are most important for the component. Variables with high absolute loadings are intercorrelated; the same sign indicate a positive intercorrelation while different signs for two variables with high loadings indicate a negative correlation. R 2 and Q 2 are reported for each significant [file 3985154.f1.docx]

Supplementary material

**Table A.** **OPLS regression of PPT using cytokines as regressors in all subjects and in healthy controls (HC).**

| **All subjects** | **VIP** | **CoeffCS** |  | **HC** | **VIP** | **CoeffCS** |
| --- | --- | --- | --- | --- | --- | --- |
| **IL-10** | 1.40 | -0.06 |  | **IL-6** | 1.69 | -0.08 |
| **IL-17A** | 1.39 | -0.06 |  | **IL-10** | 1.49 | -0.07 |
| **IL-2** | 1.36 | -0.06 |  | **IL-17A** | 1.19 | -0.05 |
| **IL-6** | 1.34 | -0.05 |  | **IFN-γ** | 1.19 | -0.06 |
| **TNF-α** | 1.26 | -0.05 |  | **IL-1ra** | 1.02 | -0.05 |
| **IFN-γ** | 1.18 | -0.05 |  | **TNF-α** | 0.96 | -0.04 |
| **eotaxin** | 0.77 | -0.03 |  | **IL-8** | 0.85 | -0.04 |
| **IP-10** | 0.70 | -0.03 |  | **IL-2** | 0.82 | -0.04 |
| **IL-1ra** | 0.54 | -0.02 |  | **IL-1β** | 0.62 | -0.03 |
| **IL-8** | 0.20 | -0.01 |  | **IP-10** | 0.53 | -0.02 |
| **MCP-1** | 0.16 | 0.01 |  | **MCP-1** | 0.25 | -0.01 |
| **IL-1β** | 0.10 | 0.003 |  | **eotaxin** | 0.03 | -0.01 |
| ***R2*** | 0.06 |  |  | ***R2*** | 0.10 |  |
| ***Q2*** | 0.05 |  |  | ***Q2*** | 0.07 |  |
| **CV-ANOVA** | 0.003 |  |  | **CV-ANOVA** | 0.009 |  |

OPLS= orthogonal partial least squares; PPT= pressure pain threshold. VIP>1.0 is significant. The sign of CoefCS shows the direction of the correlation. Thus, negative values of CoefCS indicate that this variable is low in fibromyalgia (FM) and a positive value that this variable is high in FM. The three bottom rows report *R^2^, Q^2^* and CV-ANOVA (*P*-value).

**Table B.** **OPLS regression of PDI in fibromyalgia (FM) using cytokines as regressors.**

| **Variables** | **VIP** | **CoeffCS** |
| --- | --- | --- |
| **IL-10** | 1.83 | 0.20 |
| **MCP-1** | 1.24 | -0.11 |
| **IL-1ra** | 1.23 | -0.15 |
| **eotaxin** | 1.07 | -0.09 |
| **IP-10** | 1.06 | -0.15 |
| **IL-2** | 1.01 | 0.10 |
| **IL-1β** | 0.97 | -0.19 |
| **IL-6** | 0.80 | 0.08 |
| **TNF-α** | 0.60 | 0.03 |
| **IL-17A** | 0.57 | -0.11 |
| **IFN-γ** | 0.22 | -0.01 |
| **IL-8** | 0.14 | -0.01 |
| ***R2*** | 0.19 |  |
| ***Q2*** | 0.08 |  |
| **CV-ANOVA** | 0.05 |  |

OPLS= orthogonal partial least squares; PDI= Pain Disability Index. VIP>1.0 is significant. The sign of CoefCS and *p(corr)* shows the direction of the correlation. Thus, negative values of CoeffCS indicate that this variable is low in FM and a positive value that this variable is high in FM. The three bottom rows report *R^2^, Q^2^* and CV-ANOVA (*P*-value).

**Table C. PCAs of changes in cytokines and clinical psychometric instruments in both intervention groups taken together.**

| **Variables** | **p[1]** | **p[2]** |
| --- | --- | --- |
| **ΔVAS global pain intensity** | 0.16 | **0.24** |
| **ΔPPT** | **0.21** | 0.05 |
| **ΔElbow flexion force** | 0.14 | 0.07 |
| **ΔKnee extension force** | 0.10 | 0.09 |
| **Δ6MWT** | 0.07 | 0.10 |
| **ΔHADS-depression** | 0.19 | **0.21** |
| **ΔHADS-anxiety** | 0.10 | **0.22** |
| **ΔSF-36PCS** | **0.20** | 0.14 |
| **ΔSF-36MSC** | 0.08 | **0.22** |
| **ΔFIQ** | 0.19 | **0.28** |
| **ΔMFI-general fatigue** | **0.21** | **0.23** |
| **ΔMFI-physical fatigue** | 0.18 | **0.26** |
| **ΔMFI-reduced activity** | **0.20** | **0.28** |
| **ΔMFI-reduced motivation** | 0.13 | 0.19 |
| **ΔMFI-mental fatigue** | 0.17 | 0.21 |
| **ΔIFN-γ** | **-0.33** | 0.18 |
| **ΔIL-1β** | -0.09 | -0.08 |
| **ΔIL-2** | **-0.23** | **0.28** |
| **ΔIL-6** | **-0.28** | 0.19 |
| **ΔIL-8** | **-0.28** | **0.25** |
| **ΔIL-10** | **-0.29** | **0.21** |
| **ΔIL-17A** | **-0.34** | **0.21** |
| **ΔTNF-α** | 0.03 | 0.15 |
| **ΔIL-1ra** | 0.16 | **0.24** |
| **ΔIP-10** | 0.09 | -0.01 |
| **ΔMCP-1** | 0.01 | 0.07 |
| **Δeotaxin** | -0.05 | -0.12 |
| *R^2^* | 0.19 | 0.16 |
| *Q^2^* | 0.08 | 0.10 |

PCA= Principle component analysis; Δ= difference after intervention. Two significant components (p[1] and p[2]) were obtained. For each component are reported the loadings of the variables. High absolute loadings are most important for the component. Variables with high absolute loadings are intercorrelated; same sign indicate a positive intercorrelation while different signs for two variables with high loadings indicate a negative correlation. *R^2^* and *Q^2^* are reported for each significant component at the two bottom rows. The loadings of the most important variables for the two components are denoted with bold type. See also Fig. 1. FIQ= Fibromyalgia Impact Questionnaire; HADS= Hospital Anxiety and Depression Scale; MFI= Multidimensional Fatigue Inventory; PDI= Pain Disability Index; PPT= pressure pain thresholds (mean of 8 sites); SF-36= Short Form Health Survey 36, physical (PSC) and mental summary components (MSC); 6MWT= 6-min walk test; VAS= Visual Analogue Scale.

**Table D. PCAs of baseline cytokine levels and changes in psychometric instruments in both groups taken together.**

| **Variables** | **p[1]** | **p[2]** |
| --- | --- | --- |
| **ΔVAS** **global pain intensity** | **0.25** | -0.12 |
| **ΔPPT** | 0.19 | -0.03 |
| **ΔElbow flexion force** | 0.19 | 0.05 |
| **ΔKnee extension force** | 0.18 | 0.06 |
| **Δ6MWT** | 0.08 | -0.08 |
| **ΔHADS-depression** | **0.26** | -0.12 |
| **ΔHADS-anxiety** | **0.21** | -0.08 |
| **ΔSF-36PCS** | **0.23** | -0.09 |
| **ΔSF-36MSC** | 0.11 | **-0.23** |
| **ΔFIQ** | **0.26** | **-0.22** |
| **ΔMFI-general fatigue** | **0.24** | **-0.21** |
| **ΔMFI-physical fatigue** | **0.24** | **-0.21** |
| **ΔMFI-reduced activity** | **0.25** | **-0.28** |
| **ΔMFI-reduced motivation** | 0.18 | -0.19 |
| **ΔMFI-mental fatigue** | **0.21** | **-0.20** |
| **IFN-γ** | **0.28** | **0.26** |
| **IL-1β** | 0.17 | **0.20** |
| **IL-2** | **0.20** | **0.32** |
| **IL-6** | **0.21** | **0.23** |
| **IL-8** | 0.15 | **0.31** |
| **IL-10** | 0.17 | **0.28** |
| **IL-17A** | **0.24** | **0.30** |
| **TNF-α** | -0.05 | 0.16 |
| **IL-1ra** | **0.25** | -0.12 |
| **IP-10** | -0.09 | -0.12 |
| **MCP-1** | -0.08 | -0.08 |
| **eotaxin** | 0.03 | -0.03 |
| *R2* | 0.18 | 0.14 |
| *Q2* | 0.05 | 0.07 |

PCA= Principle component analysis; Δ= difference after intervention. Two significant components (p[1] and p[2]) were obtained. For each component are reported the loadings of the variables. High absolute loadings are most important for the component. Variables with high absolute loadings are intercorrelated; same sign indicate a positive intercorrelation while different signs for two variables with high loadings indicate a negative correlation. *R^2^* and *Q^2^* are reported for each significant component at the two bottom rows. The loadings of the most important variables for the two components are denoted with bold type. FIQ= Fibromyalgia Impact Questionnaire; HADS= Hospital Anxiety and Depression Scale; MFI= Multidimensional Fatigue Inventory; PDI= Pain Disability Index; PPT= pressure pain thresholds (mean of 8 sites); SF-36= Short Form Health Survey 36, physical (PSC) and mental summary components (MSC); 6MWT= 6-min walk test; VAS= Visual Analogue Scale.
